# Formal Uncertainty and Dispersion of Single and Double Difference Models for GNSS-Based Attitude Determination

**DOI:** 10.3390/s17020408

**Published:** 2017-02-20

**Authors:** Wen Chen, Chao Yu, Danan Dong, Miaomiao Cai, Feng Zhou, Zhiren Wang, Lei Zhang, Zhengqi Zheng

**Affiliations:** 1 Engineering Center of SHMEC for Space Information and GNSS, East China Normal University, Shanghai 200241, China; wchen@sist.ecnu.edu.cn (W.C.); dndong@cs.ecnu.edu.cn (D.D.); mmcai@outlook.com (M.C.); zhoufecnu@163.com (F.Z.); wangwangzhiren@163.com (Z.W.); lzhang@cs.ecnu.edu.cn (L.Z.); zqzheng@ee.ecnu.edu.cn (Z.Z.); 2 Key Laboratory of Geographic Information Science, Ministry of Education, East China Normal University, Shanghai 200241, China; 3 Shanghai Key Laboratory of Multidimensional Information Processing, East China Normal University, Shanghai 200241, China

**Keywords:** formal uncertainty, dispersion, single difference, double difference, GNSS, common clock

## Abstract

With multi-antenna synchronized global navigation satellite system (GNSS) receivers, the single difference (SD) between two antennas is able to eliminate both satellite and receiver clock error, thus it becomes necessary to reconsider the equivalency problem between the SD and double difference (DD) models. In this paper, we quantitatively compared the formal uncertainties and dispersions between multiple SD models and the DD model, and also carried out static and kinematic short baseline experiments. The theoretical and experimental results show that under a non-common clock scheme the SD and DD model are equivalent. Under a common clock scheme, if we estimate stochastic uncalibrated phase delay (UPD) parameters every epoch, this SD model is still equivalent to the DD model, but if we estimate only one UPD parameter for all epochs or take it as a known constant, the SD (here called SD2) and DD models are no longer equivalent. For the vertical component of baseline solutions, the formal uncertainties of the SD2 model are two times smaller than those of the DD model, and the dispersions of the SD2 model are even more than twice smaller than those of the DD model. In addition, to obtain baseline solutions, the SD2 model requires a minimum of three satellites, while the DD model requires a minimum of four satellites, which makes the SD2 more advantageous in attitude determination under sheltered environments.

## 1. Introduction

Attitude determination (AD) is an important branch in navigation for both ground-based and space-based platforms. Traditionally inertial measurement units (IMUs) play a predominant role in AD due to their high short-term precision. Since the direct measurements of an IMU are position acceleration and angular acceleration, systematic error accumulation inevitably affects its long-term precision. On the other hand, the global navigation satellite system (GNSS)-based AD demonstrates stable long-term precision due to its driftless performance, although its short-term precision is not yet comparable with that of a high-end IMU. Its low cost and light maintenance make this new technique more attractive. Previous studies have indicated that the GNSS-based AD was complementary to the IMU-based AD [1,2,3,4,5], and the integration of the two techniques could build accurate and stable AD systems for both the short and long term [6,7]. Up to now, GNSS AD systems have been widely used in cars, ships, aircraft, spacecraft, military equipment and other fields [8,9,10,11].

The double difference (DD) model is the most popular in GNSS data analysis, because the nature of the DD ambiguity is integer and its corresponding ambiguity resolution is much more straightforward [12,13,14]. To promote the efficiency of the ambiguity solution and maximize the ambiguity success rate, a series of algorithms were developed. The least-squares ambiguity decorrelation adjustment (LAMBDA) method is the most widely used method due to its good performance and comprehensive underlying theory [15]. Based on this method, some efforts were conducted to improve the ambiguity resolution. Chang et al. [16] put forward several strategies such as symmetric pivoting, greedy selection, lazy transformations, and shrinking to reduce complexity of LAMBDA. Some researchers [17,18,19] have made use of some a priori information such as baseline length to provide guidance for the integer search, which consequently helps to achieve a faster search time and a higher success rate.

Although it has been proved theoretically that the non-common clock SD and DD models are equivalent in the sense of deriving exactly the same position and baseline solutions with same formal uncertainties [20], the emerging multi-antenna synchronized GNSS receiver (using a common clock) has prompted scientists to reconsider the equivalence problem, in particular for AD [20,21]. With this device, the SD between two antennas is able to eliminate both satellite and receiver clock errors simultaneously, and consequently the estimation parameters of the SD observation equation are reduced by omitting the receiver clock term. The remaining parameter which is coupled with the ambiguities is the uncalibrated phase delay (UPD). UPD includes the differences from the initial fractional phase delay, and delays caused by cable length and hardware circuits, thus it can be taken as a time-invariant estimated parameter. The integer part of the UPD parameter is merged into the ambiguity parameters [22], thus we only need to estimate the fractional part. Reference [23] presented an ambiguity solution integrating both SD and DD observables. First a noisy attitude solution is obtained from the DD model, then UPD is estimated based on the preliminary baseline components using sine and cosine transformations, finally the SD ambiguities and precise attitude measurements are resolved. Reference [24] also proposed a method using both SD and DD model simultaneously. After obtaining the ambiguity resolution from the DD model, the remaining parameters in the SD model were estimated with nonlinear least-square methods. The above attempts indicated that the common clock SD model is also applicable in AD. It is increasingly important to know how much this type of SD model can improve the accuracy for AD theoretically and practically.

Through experiments, [25] found that the SD model under a common clock scheme reduces the dispersion of pitch angle solutions from a value 0.2° for the DD model to 0.1° for 2 m baseline, whereas the dispersion of yaw angle solutions from both the SD and DD models are equivalent. Why the solution differences occur only in the pitch angle and not in the yaw angle? Although [25] did not provide theoretical proof it gave an intuitive explanation, i.e., the satellite orbits cover a full 360° range of azimuth angle but only cover half the inclination angle range (the upper hemisphere). This uneven distribution might generate different SD and DD estimates of the vertical component of baseline vector, which is related to pitch and roll angles. Reference [20] performed a theoretical investigation on AD for single epoch solutions. They proved that for a traditional GNSS receiver the SD model still estimated receiver clock parameters. In this case the formal uncertainties of baseline vector parameters are exactly same for the SD and DD models. Under the common clock scheme, however, the formal uncertainties of horizontal components from the SD and DD models are very close, while the formal uncertainty of vertical components from the SD model is notably smaller than that of the DD model. Their simulated attitude solutions demonstrated that under the common clock scheme the standard deviations of yaw angle solutions were only slightly improved (0.188° for SD vs. 0.195° for DD), but the standard deviations of pitch angle solutions were greatly improved (0.184° for SD vs. 0.419° for DD) [20]. Their research provided a theoretical framework for the equivalence problem between the SD and DD models, but their theoretical discussion was limited to formal uncertainty only. The equivalence problem for dispersion of real baseline solutions is practically important and still requires quantitative investigation and assessment. Additionally, their analysis assumed that the UPD ([20] called it line bias) was zero or negligible. However, the UPD is actually not negligible due to the initial fractional phase delay, even if the propagation delay from cables and hardware path could be omitted under ideal conditions. Thus the equivalence problem for non-zero UPD parameters should also be discussed.

This paper first revisits the equivalence problem of formal uncertainty between the SD and DD models [20]. We embody the comparison quantitatively by the statistical results. Then we discuss the statistics of dispersion between the SD and DD models. Dispersion is tied to real accuracy more closely. We further discuss the common clock SD model with UPD parameter, which is widely applied. Both static and kinematic short baseline experiments are carried out.

## 2. Theoretical Analysis

### 2.1. Comparison between SD and DD under Non-Common Clock Scheme

Following [20], we simplify the problem and assume that both the SD and DD integer ambiguities have been fixed. All SD observables are equal-weighted (let the weight be 1) and independent. By forming the SD, the satellite clock term is eliminated. For short baseline observables, the influence of atmospheric and ionospheric delays can be ignored. In addition, we only discuss measurements at L1-band and focus on static solutions, the SD measurement equation becomes:

(1) $$\Delta (L^{i}-L_{0}^{i})=A^{i}\delta b+u+\varepsilon^{i}$$

where L is the carrier phase observable (unit: meter); $L_{0}$ is the initial carrier phase observable derived from a priori information; i denotes the satellite index; Δ is the SD operator; δb is the estimated correction of baseline vector; A is the partial derivative matrix of the SD carrier phase observable respect to baseline vector. The parameter u includes the receiver clock error difference and UPD difference under the non-common clock scheme, which is the same for all satellites. ε is the SD observation error.

For single epoch, the covariance sub-matrix of the baseline vector is:

(2) $$Q_{b}=\sigma^{2}{\left(A^{T}\left(I_{n}-\frac{1}{n}e_{n}e_{n}^{T}\right)A\right)}^{-1}$$

where $\sigma^{2}$ is the variance of the observables; $e_{n}$ is n×1 unit vector; n is the number of satellites at each epoch; $I_{n}$ is n×n unit matrix. The baseline vector solution is:

(3) $$\delta \hat{b}=\frac{1}{\sigma^{2}}Q_{b}A^{T}\left(I_{n}-\frac{1}{n}e_{n}e_{n}^{T}\right)\left(\Delta \left(L^{i}-L_{0}^{i}\right)\right)$$

For the DD model, after linearization, the DD measurement equation can be expressed as:

(4) $$D\Delta \left(L-L_{0}\right)=DA\delta b+D\varepsilon$$

where *D* is the DD operator. And the least squares estimation of baseline vector is given by:

(5) $$\delta \hat{b}={\left(A^{T}D^{T}{\left(DD^{T}\right)}^{-1}DA\right)}^{-1}A^{T}D^{T}{\left(DD^{T}\right)}^{-1}\left(D\Delta \left(L-L_{0}\right)\right)$$

According to [26]:

(6) $$D^{T}{\left(DD^{T}\right)}^{-1}D=I_{n}-\frac{1}{n}e_{n}e_{n}^{T}$$

The covariance sub-matrix of the baseline vector and the baseline solutions from the DD are exactly the same as those from non-common clock SD (Equations (2) and (3), respectively). Thus the single epoch DD and SD models are equivalent under a non-common clock scheme [20].

In kinematic mode the single epoch solutions at each epoch are uncorrelated. This case is equivalent to the above single epoch case and the baseline esimations from the DD and SD models are still equivalent. In the static SD mode with multi-epoch joint estimate, the covariance matrix of the baseline vector solution is given by:

(7) $$Q_{b}=\sigma^{2}{\left(\sum_{1}^{m}A_{i}^{T}A_{i}-\sum_{1}^{m}A_{i}^{T}e_{n_{i}}{\left(e_{n_{i}}^{T}e_{n_{i}}\right)}^{-1}e_{n_{i}}^{T}A_{i}\right)}^{-1}=\sigma^{2}{\left(\sum_{1}^{m}A_{i}^{T}\left(I_{n_{i}}-\frac{1}{n_{i}}e_{n_{i}}e_{n_{i}}^{T}\right)A_{i}\right)}^{-1}$$

where i is epoch index; m is the total number of epochs. The baseline vector solution is:

(8) $$\delta \hat{b}=\frac{1}{\sigma^{2}}Q_{b}\sum_{1}^{m}A_{i}^{T}\left(I_{n_{i}}-\frac{1}{n_{i}}e_{n_{i}}e_{n_{i}}^{T}\right)\left(\Delta \left(L_{i}-L_{0i}\right)\right)$$

With the same method used above, it can be proved that the static multi-epoch DD and SD models under a non-common clock scheme are also equivalent. Owing to this equivalency, in the following sections we use the non-common clock SD model to represent the DD model for comparison.

### 2.2. Comparison between Non-Common Clock SD and Common Clock SD without UPD

Under the common clock scheme, the receiver clock error is canceled out by forming SD, thus only UPD is included in parameter *u*. [20] provided the comparison of formal uncertainty expressions between the non-common clock SD and the common clock SD without UPD (UPD = 0). Assume UPD = 0, the measurement equation of the common clock SD is:

(9) $$\Delta (L^{i}-L_{0}^{i})=A^{i}\delta b+\varepsilon^{i}$$

The baseline vector solution and its covariance matrix are:

(10) $$\delta \tilde{b}=\frac{1}{\sigma^{2}}Q_{\tilde{b}}A^{T}\left(\Delta \left(L-L_{0}\right)\right)$$

(11) $$Q_{\tilde{b}}=\sigma^{2}{\left(A^{T}A\right)}^{-1}$$

For comparison, we rewrite the Equation (2) of the non-common clock SD model:

(12) $$\sigma^{2}{\left(A^{T}\left(I-\frac{1}{n}e_{n}e_{n}^{T}\right)A\right)}^{-1}=\sigma^{2}{\left(M-vv^{T}\right)}^{-1}$$

where $M=A^{T}A,\;v=\frac{1}{\sqrt{n}}A^{T}e_{n}$. With the matrix inversion lemma, we have:

(13) $${\left(M-vv^{T}\right)}^{-1}=M^{-1}+\frac{1}{1-v^{T}M^{-1}v}\left(M^{-1}v\right){\left(M^{-1}v\right)}^{T}$$

It’s easy to find that the first term on the right side of Equation (13) is the same as the baseline solution covariance matrix of the common clock SD model (Equation (11)) and the second term represents the formal uncertainty difference between the non-common clock SD model and common clock SD model without UPD. From the theoretical discussion of [20], the second term of Equation (13) is always positive, hence the formal uncertainty from the non-common clock SD scheme is always larger than that from the common clock SD scheme without UPD. Geometrical analysis indicates that the difference of formal uncertainty is distributed primarily in the up direction. Here we further evaluate the differences of baseline solutions between the non-common clock SD scheme and the common clock SD scheme without UPD.

Substituting Equations (9) and (11) into Equation (10), we obtain:

(14) $$\delta \tilde{b}=\delta b+{\left(A^{T}A\right)}^{-1}A^{T}\varepsilon$$

Also, substituting Equations (1) and (2) into Equation (3), the estimation of baseline vector is:

(15) $$\delta \hat{b}=\delta b+{\left(A^{T}\left(I_{n}-\frac{1}{n}e_{n}e_{n}^{T}\right)A\right)}^{-1}A^{T}\left(I_{n}-\frac{1}{n}e_{n}e_{n}^{T}\right)\left(u+\varepsilon \right)$$

where δb is the true value of baseline solution. The second term on the right side of Equation (14) shows that the deviation of the baseline estimation from the common clock SD is only related to the SD observation errors ε. If we regard ${\left(A^{T}A\right)}^{-1}$ as the weight matrix, the deviation of baseline solution is the weighted average of ε. In contrast for the non-common clock SD the deviation (the second term on the right side of Equation (15)) can be taken as the weighted average of parameter *u* (receiver clock error and UPD) and observation errors. Since both the receiver clock error and UPD are satellite independent, it can be proved that:

(16) $$\left(I_{n}-\frac{1}{n}e_{n}e_{n}^{T}\right)u=\left(\begin{array}{cccc} 1-\frac{1}{n} & -\frac{1}{n} & \cdots & -\frac{1}{n}\\ -\frac{1}{n} & 1-\frac{1}{n} & \cdots & -\frac{1}{n}\\ \vdots & \vdots & \ddots & \vdots \\ -\frac{1}{n} & -\frac{1}{n} & \cdots & 1-\frac{1}{n} \end{array}\right)\left[\begin{array}{c} 1\\ 1\\ \vdots \\ 1 \end{array}\right]u=\left[\begin{array}{c} 0\\ 0\\ \vdots \\ 0 \end{array}\right]$$

Then the parameter *u* can be omitted from Equation (15). Hence, both the estimated solutions (Equations (14) and (15)) are unbiased estimations of the true value, but their dispersions of solutions are different.

For convenience, the baseline vector parameters are expressed in a local geographical coordinate system (east, north and up), the corresponding partial derivative matrix $A^{i}$ for satellite *i* is also expressed in the same coordinate system:

(17) $$A^{i}=\left(\begin{array}{ccc} \frac{x_{e}^{i}-x_{e}}{\rho^{i}} & \frac{x_{n}^{i}-x_{n}}{\rho^{i}} & \frac{x_{u}^{i}-x_{u}}{\rho^{i}} \end{array}\right)=\left(\begin{array}{ccc} \cos\theta_{e}^{i} & \cos\theta_{n}^{i} & \cos\theta_{u}^{i} \end{array}\right)$$

where $\rho^{i}$ is the geometric distance between the *i*-th satellite and antenna; $x^{i}-x$ are three components of the line from the *i*-th satellite to the antenna in the local geographical coordinate system. We assume that the priori position vector of the antenna is close to the true value. θ denotes the angle between satellite-antenna vector and the axes of the local geographical coordinate system.

Compared with the true value, the deviations of baseline vector solution in Equations (14) and (15) can be regarded as stochastic variables, and are written respectively as:

(18) $$\left[\begin{array}{c} \Delta b_{e}\\ \Delta b_{n}\\ \Delta b_{u} \end{array}\right]={\left[\begin{array}{ccc} \sum_{i}{\left(\cos\theta_{e}^{i}\right)}^{2} & \sum_{i}\cos\theta_{e}^{i}\cos\theta_{n}^{i} & \sum_{i}\cos\theta_{e}^{i}\cos\theta_{u}^{i}\\ \sum_{i}\cos\theta_{n}^{i}\cos\theta_{e}^{i} & \sum_{i}{\left(\cos\theta_{n}^{i}\right)}^{2} & \sum_{i}\cos\theta_{n}^{i}\cos\theta_{u}^{i}\\ \sum_{i}\cos\theta_{u}^{i}\cos\theta_{e}^{i} & \sum_{i}\cos\theta_{u}^{i}\cos\theta_{n}^{i} & \sum_{i}{\left(\cos\theta_{u}^{i}\right)}^{2} \end{array}\right]}^{-1}\left[\begin{array}{c} \sum \cos\theta_{e}^{i}\varepsilon^{i}\\ \sum \cos\theta_{n}^{i}\varepsilon^{i}\\ \sum \cos\theta_{u}^{i}\varepsilon^{i} \end{array}\right]$$

(19) $$\left[\begin{array}{c} \Delta b_{e}\\ \Delta b_{n}\\ \Delta b_{u}\\ \Delta u \end{array}\right]={\left(\begin{array}{cccc} \sum_{i}{\left(\cos\theta_{e}^{i}\right)}^{2} & \sum_{i}\cos\theta_{e}^{i}\cos\theta_{n}^{i} & \sum_{i}\cos\theta_{e}^{i}\cos\theta_{u}^{i} & \sum_{i}\cos\theta_{e}^{i}\\ \sum_{i}\cos\theta_{n}^{i}\cos\theta_{e}^{i} & \sum_{i}{\left(\cos\theta_{n}^{i}\right)}^{2} & \sum_{i}\cos\theta_{n}^{i}\cos\theta_{u}^{i} & \sum_{i}\cos\theta_{n}^{i}\\ \sum_{i}\cos\theta_{u}^{i}\cos\theta_{e}^{i} & \sum_{i}\cos\theta_{u}^{i}\cos\theta_{n}^{i} & \sum_{i}{\left(\cos\theta_{u}^{i}\right)}^{2} & \sum_{i}\cos\theta_{u}^{i}\\ \sum_{i}\cos\theta_{e}^{i} & \sum_{i}\cos\theta_{n}^{i} & \sum_{i}\cos\theta_{u}^{i} & n \end{array}\right)}^{-1}\left[\begin{array}{c} \sum \cos\theta_{e}^{i}\varepsilon^{i}\\ \sum \cos\theta_{n}^{i}\varepsilon^{i}\\ \sum \cos\theta_{u}^{i}\varepsilon^{i}\\ \sum \varepsilon^{i} \end{array}\right]$$

Equations (18) and (19) represent the deviations of the baseline vector solution from the true value in the common clock SD without UPD and the non-common clock SD schemes, respectively. However, it is difficult to derive the analytical formula of the inverse of the normal matrix in these two equations. To get the impression of the dispersion differences between the two models intuitively, we discuss their asymptotic case. Assume that the number of satellites is extremely abundant (that is n→∞), the occurrences of satellites in the upper hemisphere are uniformly distributed, and the satellite positions in the sky are independent, then the summation matrix elements in Equations (18) and (19) can be expressed using their mathematical expectations, hence:

(20) $$\begin{array}{ll} E\left\{\cos\theta_{n}^{i}\right\} & =E\left\{\cos\theta_{e}^{i}\right\}=E\left\{\cos\theta_{e}^{i}\cos\theta_{n}^{i}\right\}=E\left\{\cos\theta_{e}^{i}\cos\theta_{u}^{i}\right\}=E\left\{\cos\theta_{n}^{i}\cos\theta_{u}^{i}\right\}=0\\ E\left\{\cos\theta_{u}^{i}\right\} & =\iint \cos\theta_{u}\sin\theta_{u}d\theta_{u}d\lambda /\iint \sin\theta_{u}d\theta_{u}d\lambda \\ & =2\pi \int_{0}^{\frac{\pi }{2}}\cos\theta_{u}\sin\theta_{u}d\theta_{u}/2\pi =\frac{1}{2}\\ E\left\{\cos^{2}\theta_{e}^{i}\right\} & =\iint \cos^{2}\theta_{e}\sin\theta_{u}d\theta_{u}d\lambda /\iint \sin\theta_{u}d\theta_{u}d\lambda \\ & =\int_{0}^{2\pi }\int_{0}^{\frac{\pi }{2}}\left(\cos^{2}\lambda \sin^{3}\theta_{u}\right)d\theta_{u}d\lambda /2\pi =\frac{1}{2}\left(1-\frac{1}{3}\right)=\frac{1}{3}\\ E\left\{\cos^{2}\theta_{n}^{i}\right\} & =E\left\{\cos^{2}\theta_{e}^{i}\right\}=\frac{1}{3}\\ E\left\{\cos^{2}\theta_{u}^{i}\right\} & =\iint \cos^{2}\theta_{u}\sin\theta_{u}d\theta_{u}d\lambda /\iint \sin\theta_{u}d\theta_{u}d\lambda \\ & =2\pi \int_{0}^{\frac{\pi }{2}}-\cos^{2}\theta_{u}d\cos\theta_{u}/2\pi =\frac{1}{3} \end{array}$$

Then, the statistical result of covariance matrix of the baseline solution from the common clock SD model without UPD is:

(21) $$\sigma^{2}{\left[\begin{array}{ccc} \frac{n}{3} & 0 & 0\\ 0 & \frac{n}{3} & 0\\ 0 & 0 & \frac{n}{3} \end{array}\right]}^{-1}=\sigma^{2}\left[\begin{array}{ccc} \frac{3}{n} & 0 & 0\\ 0 & \frac{3}{n} & 0\\ 0 & 0 & \frac{3}{n} \end{array}\right]$$

While that from the non-common clock SD gives:

(22) $$\sigma^{2}{\left(\begin{array}{cccc} \frac{n}{3} & 0 & 0 & 0\\ 0 & \frac{n}{3} & 0 & 0\\ 0 & 0 & \frac{n}{3} & \frac{n}{2}\\ 0 & 0 & \frac{n}{2} & n \end{array}\right)}^{-1}=\sigma^{2}\left(\begin{array}{cccc} \frac{3}{n} & 0 & 0 & 0\\ 0 & \frac{3}{n} & 0 & 0\\ 0 & 0 & \frac{12}{n} & \frac{-6}{n}\\ 0 & 0 & \frac{-6}{n} & \frac{4}{n} \end{array}\right)$$

Statistically, the formal uncertainties of the horizontal baseline components from the two SD models are equal (corresponding to the yaw angle), and the formal uncertainty of the vertical baseline component (corresponding to the pitch angle) from the common clock SD model without UPD is also equivalent to that of horizontal component, whereas the formal uncertainty of vertical component from the non-common clock SD model is twice as large as that of the horizontal component. These conclusions agree with the results of [20]. We also give the quantitative asymptotic difference of the vertical formal uncertainty between the two SD schemes, i.e., in the non-common clock SD scheme the vertical uncertainty is twice as large as that in the common clock SD case. Its geometrical cause stems from the uneven satellite distribution in elevation angle. Since the orbital distribution only covers the upper hemisphere, the average value of satellite elevations is not zero, hence the vertical baseline component is correlated with the parameter *u*.

We now consider the dispersion of baseline solutions. In statistics, the deviation of baseline solution from the common clock SD model without UPD can be denoted by:

(23) $$\left[\begin{array}{c} \Delta b_{e}\\ \Delta b_{n}\\ \Delta b_{u} \end{array}\right]=\left[\begin{array}{ccc} \frac{3}{n} & 0 & 0\\ 0 & \frac{3}{n} & 0\\ 0 & 0 & \frac{3}{n} \end{array}\right]\left[\begin{array}{c} \sum \cos\theta_{e}^{i}\varepsilon^{i}\\ \sum \cos\theta_{n}^{i}\varepsilon^{i}\\ \sum \cos\theta_{u}^{i}\varepsilon^{i} \end{array}\right]$$

According to the variance formula for the multiplication of two independent stochastic variables:

(24) $$V(xy)=V(x)\cdot V(y)+V(x)\cdot {\left(E(y)\right)}^{2}+V(y)\cdot {\left(E(x)\right)}^{2}$$

(25) $$V\left[\begin{array}{c} \Delta b_{e}\\ \Delta b_{n}\\ \Delta b_{u} \end{array}\right]=\left[\begin{array}{ccc} \frac{3}{n} & 0 & 0\\ 0 & \frac{3}{n} & 0\\ 0 & 0 & \frac{3}{n} \end{array}\right]\left[\begin{array}{ccc} \frac{n\sigma^{2}}{3} & 0 & 0\\ 0 & \frac{n\sigma^{2}}{3} & 0\\ 0 & 0 & \frac{n\sigma^{2}}{3} \end{array}\right]\left[\begin{array}{ccc} \frac{3}{n} & 0 & 0\\ 0 & \frac{3}{n} & 0\\ 0 & 0 & \frac{3}{n} \end{array}\right]=\left[\begin{array}{ccc} \frac{3}{n} & 0 & 0\\ 0 & \frac{3}{n} & 0\\ 0 & 0 & \frac{3}{n} \end{array}\right]\sigma^{2}$$

here *V* denotes the variance operator. Similarly, the deviation of baseline solution from non-common clock SD is:

(26) $$\left[\begin{array}{c} \Delta b_{e}\\ \Delta b_{n}\\ \Delta b_{u}\\ \Delta u \end{array}\right]=\left(\begin{array}{cccc} \frac{3}{n} & 0 & 0 & 0\\ 0 & \frac{3}{n} & 0 & 0\\ 0 & 0 & \frac{12}{n} & \frac{-6}{n}\\ 0 & 0 & \frac{-6}{n} & \frac{4}{n} \end{array}\right)\left[\begin{array}{c} \sum \cos\theta_{e}^{i}\varepsilon^{i}\\ \sum \cos\theta_{n}^{i}\varepsilon^{i}\\ \sum \cos\theta_{u}^{i}\varepsilon^{i}\\ \sum \varepsilon^{i} \end{array}\right]$$

(27) $$V\left[\begin{array}{c} \Delta b_{e}\\ \Delta b_{n}\\ \Delta b_{u}\\ \Delta u \end{array}\right]=\left(\begin{array}{cccc} \frac{3}{n} & 0 & 0 & 0\\ 0 & \frac{3}{n} & 0 & 0\\ 0 & 0 & \frac{12}{n} & \frac{-6}{n}\\ 0 & 0 & \frac{-6}{n} & \frac{4}{n} \end{array}\right)\left[\begin{array}{cccc} \frac{n\sigma^{2}}{3} & 0 & 0 & 0\\ 0 & \frac{n\sigma^{2}}{3} & 0 & 0\\ 0 & 0 & \frac{n\sigma^{2}}{3} & 0\\ 0 & 0 & 0 & n\sigma^{2} \end{array}\right]\left(\begin{array}{cccc} \frac{3}{n} & 0 & 0 & 0\\ 0 & \frac{3}{n} & 0 & 0\\ 0 & 0 & \frac{12}{n} & \frac{-6}{n}\\ 0 & 0 & \frac{-6}{n} & \frac{4}{n} \end{array}\right)=\left(\begin{array}{cccc} \frac{3}{n} & 0 & 0 & 0\\ 0 & \frac{3}{n} & 0 & 0\\ 0 & 0 & \frac{84}{n} & \frac{-48}{n}\\ 0 & 0 & \frac{-48}{n} & \frac{28}{n} \end{array}\right)\sigma^{2}$$

Comparing Equation (25) with Equation (27), statistically, the dispersions of the horizontal component (correspondence to the yaw angle) from both SD models are still equivalent, the dispersion of vertical component from the common clock SD without UPD is also equal to that of the horizontal component, whereas the dispersion of the vertical component from the non-common clock SD is 5.3 times as large as the horizontal component, which is also much more than that of the vertical component from the common clock SD without UPD.

The above discussion has focused on the case of single epoch baseline solutions. For the static multi-epoch case, the baseline solutions for the two SD models are:

(28) $$\left[\begin{array}{c} \Delta b_{e}\\ \Delta b_{n}\\ \Delta b_{u} \end{array}\right]={\left[\begin{array}{ccc} \sum_{ij}{\left(\cos\theta_{e}^{ij}\right)}^{2} & \sum_{ij}\cos\theta_{e}^{ij}\cos\theta_{n}^{ij} & \sum_{ij}\cos\theta_{e}^{ij}\cos\theta_{u}^{ij}\\ \sum_{ij}\cos\theta_{n}^{ij}\cos\theta_{e}^{ij} & \sum_{ij}{\left(\cos\theta_{n}^{ij}\right)}^{2} & \sum_{ij}\cos\theta_{n}^{ij}\cos\theta_{u}^{ij}\\ \sum_{ij}\cos\theta_{u}^{ij}\cos\theta_{e}^{ij} & \sum_{ij}\cos\theta_{u}^{ij}\cos\theta_{n}^{ij} & \sum_{ij}{\left(\cos\theta_{u}^{ij}\right)}^{2} \end{array}\right]}^{-1}\left[\begin{array}{c} \sum \cos\theta_{e}^{ij}\varepsilon^{ij}\\ \sum \cos\theta_{n}^{ij}\varepsilon^{ij}\\ \sum \cos\theta_{u}^{ij}\varepsilon^{ij} \end{array}\right]$$

(29) $$\left[\begin{array}{c} \Delta b_{e}\\ \Delta b_{n}\\ \Delta b_{u}\\ \Delta u_{1}\\ \vdots \\ \Delta u_{m} \end{array}\right]={\left[\begin{array}{cccccc} \sum_{ij}{\left(\cos\theta_{e}^{ij}\right)}^{2} & \sum_{ij}\cos\theta_{e}^{ij}\cos\theta_{n}^{ij} & \sum_{ij}\cos\theta_{e}^{ij}\cos\theta_{u}^{ij} & \sum_{i}\cos\theta_{e}^{i1} & \cdots & \sum_{i}\cos\theta_{e}^{im}\\ \sum_{ij}\cos\theta_{n}^{ij}\cos\theta_{e}^{ij} & \sum_{ij}{\left(\cos\theta_{n}^{ij}\right)}^{2} & \sum_{ij}\cos\theta_{n}^{ij}\cos\theta_{u}^{ij} & \sum_{i}\cos\theta_{n}^{i1} & \cdots & \sum_{i}\cos\theta_{n}^{im}\\ \sum_{ij}\cos\theta_{u}^{ij}\cos\theta_{e}^{ij} & \sum_{ij}\cos\theta_{u}^{ij}\cos\theta_{n}^{ij} & \sum_{ij}{\left(\cos\theta_{u}^{ij}\right)}^{2} & \sum_{i}\cos\theta_{u}^{i1} & \cdots & \sum_{i}\cos\theta_{u}^{im}\\ \sum_{i}\cos\theta_{e}^{i1} & \sum_{i}\cos\theta_{n}^{i1} & \sum_{i}\cos\theta_{u}^{i1} & n & \cdots & 0\\ \vdots & \vdots & \vdots & \vdots & \ddots & 0\\ \sum_{i}\cos\theta_{e}^{im} & \sum_{i}\cos\theta_{n}^{im} & \sum_{i}\cos\theta_{u}^{im} & 0 & \cdots & n \end{array}\right]}^{-1}\left[\begin{array}{c} \sum_{ij}\cos\theta_{e}^{ij}\varepsilon^{ij}\\ \sum_{ij}\cos\theta_{n}^{ij}\varepsilon^{ij}\\ \sum_{ij}\cos\theta_{u}^{ij}\varepsilon^{ij}\\ \sum_{i}\varepsilon^{i1}\\ \vdots \\ \sum_{i}\varepsilon^{im} \end{array}\right]$$

where i is the satellite index. For simplicity, we assume that the number of satellites in each epoch is the same (n satellites), *j* is the index of epoch (m epochs in all). 

Then, covariance matrix from common clock SD model without UPD is:

(30) $$\sigma^{2}{\left[\begin{array}{ccc} \frac{nm}{3} & 0 & 0\\ 0 & \frac{nm}{3} & 0\\ 0 & 0 & \frac{nm}{3} \end{array}\right]}^{-1}=\sigma^{2}\left[\begin{array}{ccc} \frac{3}{nm} & 0 & 0\\ 0 & \frac{3}{nm} & 0\\ 0 & 0 & \frac{3}{nm} \end{array}\right]$$

Whereas that from the non-common clock SD model is:

(31) $$\sigma^{2}{\left[\begin{array}{cccccc} \frac{nm}{3} & 0 & 0 & 0 & \cdots & 0\\ 0 & \frac{nm}{3} & 0 & 0 & \cdots & 0\\ 0 & 0 & \frac{nm}{3} & \frac{n}{2} & \cdots & \frac{n}{2}\\ 0 & 0 & \frac{n}{2} & n & \cdots & 0\\ \vdots & \vdots & \vdots & \vdots & \ddots & \vdots \\ 0 & 0 & \frac{n}{2} & 0 & \cdots & n \end{array}\right]}^{-1}=\sigma^{2}\left[\begin{array}{cccccc} \frac{3}{nm} & 0 & 0 & 0 & \cdots & 0\\ 0 & \frac{3}{nm} & 0 & 0 & \cdots & 0\\ 0 & 0 & \frac{12}{nm} & \frac{-6}{nm} & \cdots & \frac{-6}{nm}\\ 0 & 0 & \frac{-6}{nm} & \frac{m+3}{nm} & \cdots & \frac{3}{nm}\\ \vdots & \vdots & \vdots & \vdots & \ddots & \vdots \\ 0 & 0 & \frac{-6}{nm} & \frac{3}{nm} & \cdots & \frac{m+3}{nm} \end{array}\right]$$

The dispersion equations can be obtained in a similar way, so their description is omitted. It shows that in multi-epoch static case the proportional relationship of formal uncertainties between horizontal and vertical components is similar to single epoch static case, and the same for dispersion. Since nm≫n, the formal uncertainties and dispersions of the multi-epoch static solutions are smaller than those of single epoch solutions. 

Such proportional relationships of formal uncertainties and dispersions can also be extended to the multi-epoch kinematic case. For simplicity, we take two epochs for example. As for the common clock SD model without UPD, both Equations (21) and (25) become a 6 × 6 matrix which consists of two 3 × 3 diagonal sub-matrices like Equations (21) and (25), thus its formal uncertainty and dispersion are the same as those of the single epoch model. For the non-common clock SD, both Equations (22) and (27) become an 8 × 8 matrix which consists of two 4 × 4 diagonal sub-matrices like Equations (22) and (27), and its results are still the same as those of the single epoch model. 

Based on the above discussions the following conclusions are reached: both for the single epoch and the multi-epoch solution, both in static and kinematic mode, the formal uncertainty and dispersion of horizontal baseline component from the non-common clock SD scheme (and DD) is equivalent to those from a common clock SD scheme without UPD. For the vertical baseline component, however, the formal uncertainty and dispersion from the common clock SD scheme are still the same as the horizontal baseline components, but those from the non-common SD scheme model are much greater than those from the common clock SD scheme without UPD. 

### 2.3. Common Clock SD with UPD Estimated and Constrained

Although the receiver clock error can be eliminated by the SD under a common clock scheme, the UPD difference still remains in parameter *u*. For all the satellites the UPD differences are the same, otherwise the DD ambiguities are no longer integers [27]. In practice we still need to estimate the parameter *u* (or UPD) in a common clock SD scheme. Since the integer part of the parameter *u* is included in the integer ambiguities, which are assumed to be fixed already, so the range of parameter *u* should be within half a wavelength. If we impose a constraint of a half wavelength on parameters *u*, then Equation (2) becomes:

(32) $$Q_{b}=\sigma^{2}{\left(A^{T}\left(I-\frac{1}{n+\frac{4\sigma^{2}}{\lambda^{2}}}e_{n}e_{n}^{T}\right)A\right)}^{-1}$$

Usually, the measurement error of the SD carrier phase (GPS L1 band) is within 5 mm, and the UPD constraint is 100 mm, so we have $4\sigma^{2}/\lambda^{2}\approx 1/400\ll n$. Thus in the single epoch solution case, if we estimate the parameter UPD at each epoch with 0.5 wavelength constraint, Equations (32) and (2) will be very close. Similarly, if the constraint on UPD is 0.5 wavelength, the solutions of the common clock SD scheme are very close to those of the non-common clock SD and DD scheme. That means the constraint on *u* has a very small influence on the covariance matrix.

Next, we discuss the kinematic multi-epoch solution case. For simplicity, we treat UPD as a time invariant parameter with no constraint and suppose the satellite number at each epoch is the same (e.g., *n*). Note that in the previous single epoch solution case we estimate many UPD parameters for each epoch, and in this case we estimate only one UPD parameter for all epochs. For m epochs the statistical result of the covariance matrix is:

(33) $$\sigma^{2}{\left[\begin{array}{cccccccc} \frac{n}{3} & 0 & 0 & 0 & 0 & 0 & \cdots & 0\\ 0 & \frac{n}{3} & 0 & 0 & 0 & 0 & \cdots & 0\\ 0 & 0 & \frac{n}{3} & 0 & 0 & 0 & \cdots & \frac{n}{2}\\ 0 & 0 & 0 & \frac{n}{3} & 0 & 0 & \cdots & 0\\ 0 & 0 & 0 & 0 & \frac{n}{3} & 0 & \cdots & 0\\ 0 & 0 & 0 & 0 & 0 & \frac{n}{3} & \cdots & \frac{n}{2}\\ \vdots & \vdots & \vdots & \vdots & \vdots & \vdots & \ddots & \vdots \\ 0 & 0 & \frac{n}{2} & 0 & 0 & \frac{n}{2} & \cdots & nm \end{array}\right]}^{-1}=\sigma^{2}\left[\begin{array}{cccccccc} \frac{3}{n} & 0 & 0 & 0 & 0 & 0 & \cdots & 0\\ 0 & \frac{3}{n} & 0 & 0 & 0 & 0 & \cdots & 0\\ 0 & 0 & \frac{3(m+3)}{nm} & 0 & 0 & \frac{9}{nm} & \cdots & \frac{-6}{nm}\\ 0 & 0 & 0 & \frac{3}{n} & 0 & 0 & \cdots & 0\\ 0 & 0 & 0 & 0 & \frac{3}{n} & 0 & \cdots & 0\\ 0 & 0 & \frac{9}{nm} & 0 & 0 & \frac{3(m+3)}{nm} & \cdots & \frac{-6}{nm}\\ \vdots & \vdots & \vdots & \vdots & \vdots & \vdots & \ddots & \vdots \\ 0 & 0 & \frac{-6}{nm} & 0 & 0 & \frac{-6}{nm} & \cdots & \frac{4}{nm} \end{array}\right]$$

Equation (33) reveals an interesting phenomenon. In the first epoch (*m* = 1), the covariance of the vertical component is 12/n, which is equivalent to Equation (22). As the epoch number increases, the covariance of the vertical component tends to 3/*n*, which is equivalent to Equation (21). Similar to Equations (26) and (27), we calculate the statistical dispersion expression (Equation (34)), which indicates that when the observation number *m* is large, the dispersion of the vertical component will approach the dispersion of the horizontal components, similar to the common clock SD without UPD case. This multi-epoch case actually represents the kinematic AD case:

(34) $$V\left[\begin{array}{c} \Delta b_{e1}\\ \Delta b_{n1}\\ \Delta b_{u1}\\ \Delta b_{e2}\\ \Delta b_{n2}\\ \Delta b_{u2}\\ \vdots \\ \Delta u \end{array}\right]=\left[\begin{array}{cccccccc} \frac{3}{n} & 0 & 0 & 0 & 0 & 0 & \cdots & 0\\ 0 & \frac{3}{n} & 0 & 0 & 0 & 0 & \cdots & 0\\ 0 & 0 & \frac{3m^{2}+45m+36}{nm^{2}} & 0 & 0 & \frac{81}{nm} & \cdots & \frac{-48}{nm}\\ 0 & 0 & 0 & \frac{3}{n} & 0 & 0 & \cdots & 0\\ 0 & 0 & 0 & 0 & \frac{3}{n} & 0 & \cdots & 0\\ 0 & 0 & \frac{81}{nm} & 0 & 0 & \frac{3m^{2}+45m+36}{nm^{2}} & \cdots & \frac{-48}{nm}\\ \vdots & \vdots & \vdots & \vdots & \vdots & \vdots & \ddots & \vdots \\ 0 & 0 & \frac{-48}{nm} & 0 & 0 & \frac{-48}{nm} & \cdots & \frac{28}{nm} \end{array}\right]\sigma^{2}$$

Finally, we discuss the multi-epoch static case, which treats both the baseline vector and UPD as time invariant parameters, the solutions are:

(35) $$\left[\begin{array}{c} \Delta b_{e}\\ \Delta b_{n}\\ \Delta b_{u}\\ \Delta u \end{array}\right]={\left(\begin{array}{cccc} \sum_{ij}{\left(\cos\theta_{e}^{ij}\right)}^{2} & \sum_{ij}\cos\theta_{e}^{ij}\cos\theta_{n}^{ij} & \sum_{ij}\cos\theta_{e}^{ij}\cos\theta_{u}^{ij} & \sum_{ij}\cos\theta_{e}^{ij}\\ \sum_{ij}\cos\theta_{n}^{ij}\cos\theta_{e}^{ij} & \sum_{ij}{\left(\cos\theta_{n}^{ij}\right)}^{2} & \sum_{ij}\cos\theta_{n}^{ij}\cos\theta_{u}^{ij} & \sum_{ij}\cos\theta_{n}^{ij}\\ \sum_{ij}\cos\theta_{u}^{ij}\cos\theta_{e}^{ij} & \sum_{ij}\cos\theta_{u}^{ij}\cos\theta_{n}^{ij} & \sum_{ij}{\left(\cos\theta_{u}^{ij}\right)}^{2} & \sum_{ij}\cos\theta_{u}^{ij}\\ \sum_{ij}\cos\theta_{e}^{ij} & \sum_{ij}\cos\theta_{n}^{ij} & \sum_{ij}\cos\theta_{u}^{ij} & n\cdot m \end{array}\right)}^{-1}\left[\begin{array}{c} \sum \cos\theta_{e}^{i}\varepsilon^{i}\\ \sum \cos\theta_{n}^{ij}\varepsilon^{ij}\\ \sum \cos\theta_{u}^{ij}\varepsilon^{ij}\\ \sum \varepsilon^{ij} \end{array}\right]$$

The asymptotic covariance matrix is:

(36) $$\sigma^{2}{\left(\begin{array}{cccc} \frac{nm}{3} & 0 & 0 & 0\\ 0 & \frac{nm}{3} & 0 & 0\\ 0 & 0 & \frac{nm}{3} & \frac{nm}{2}\\ 0 & 0 & \frac{nm}{2} & nm \end{array}\right)}^{-1}=\sigma^{2}\left(\begin{array}{cccc} \frac{3}{nm} & 0 & 0 & 0\\ 0 & \frac{3}{nm} & 0 & 0\\ 0 & 0 & \frac{12}{nm} & \frac{-6}{nm}\\ 0 & 0 & \frac{-6}{nm} & \frac{4}{nm} \end{array}\right)$$

The proportional covariance relationship between the vertical and horizontal components is similar to Equation (22) except the n is replaced by nm. For the vertical component dispersion, we can also get the similar proportional relationship between the vertical and horizontal components like Equation (27), except the n is replaced by nm.

## 3. Experiments

Two short baseline field experiments (one static and one kinematic) were conducted to compare the performance of the SD and DD models in AD. In these experiments, a multi-antenna synchronized GNSS receiver—a Trimble BD982 GNSS receiver—was used to connect two antennas. Thus the SD observations between the two antennas are able to eliminate clock errors from both the satellite and receiver simultaneously. All SD integer ambiguities have been resolved in advance. Since the integer part of the SD UPD parameter is merged into the ambiguity parameters, the fractional part of the UPD is estimated. The DD integer ambiguities are then obtained and fixed by differencing between satellite integer SD ambiguities. The single epoch (SE) algorithm is adopt for both the SD and DD models. All results are obtained using the software called Real-time Positioning and Attitude Determination (RPAD) developed at East China Normal University. In all the experiments only the GPS L1 carrier phase observables are used.

### 3.1. Static Roof Test

**Figure 1:**
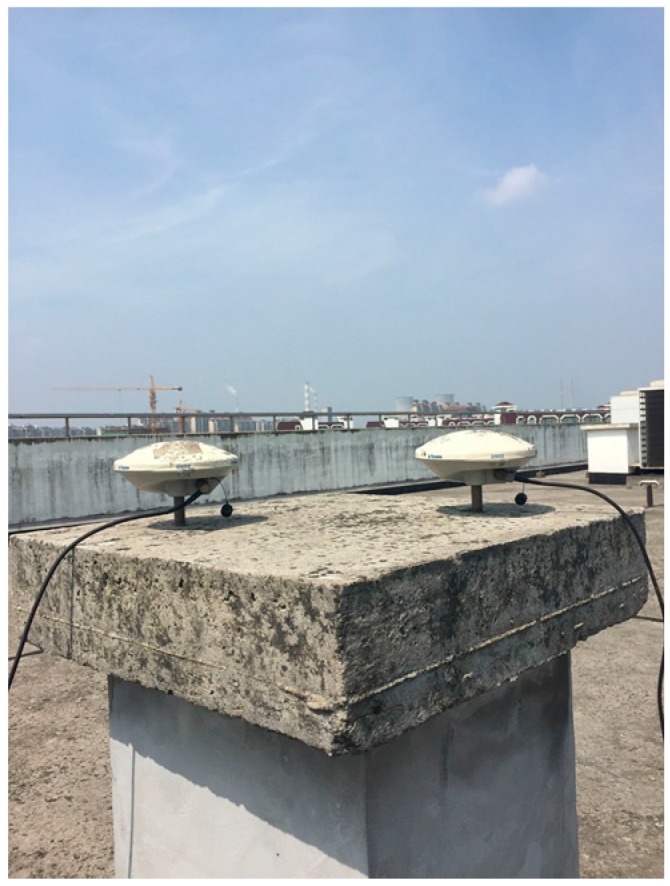
Antenna configuration in the roof test.

**Figure 2:**
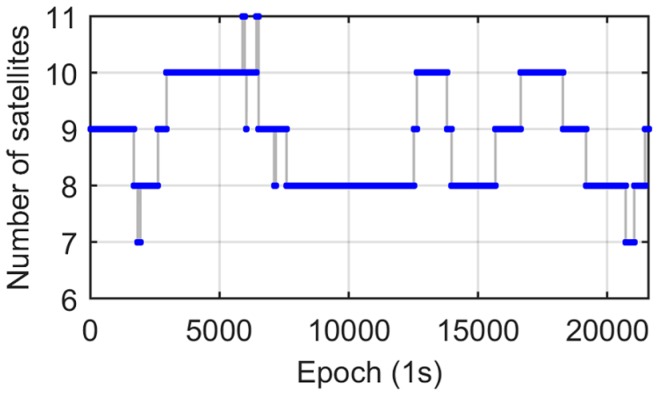
Number of visible satellites.

Two antennas were mounted on a concrete pillar on the roof of a building of the East China Normal University campus (Figure 1). The baseline length was about 398 mm. The roof test lasted about 6 hours with 1 Hz sampling rate. During the test, the visibility of satellites was fairly good. As shown in Figure 2, the number of available satellites was greater than or equal to eight most of the time, and the time with less than eight satellites only lasted 329 s.

**Figure 3:**
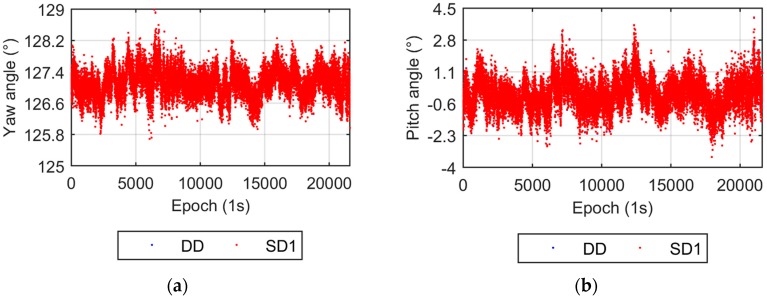
Yaw and pitch angles from the DD and SD1 models. (**a**) Yaw angles; (**b**) Pitch angles.

**Figure 4:**
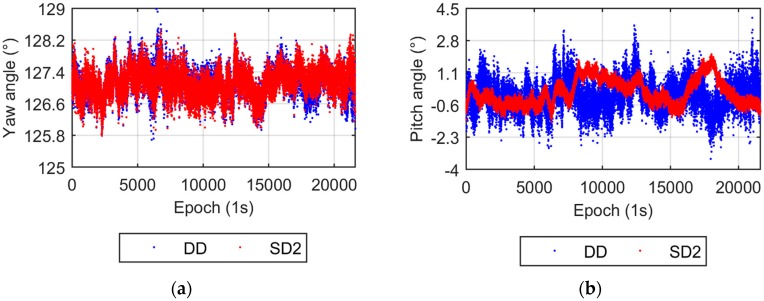
Yaw and pitch angles from the DD and SD2 models. (**a**) Yaw angles; (**b**) Pitch angles.

The UPD parameter is eliminated in the DD model, while it is estimated in the SD model. Two UPD scenarios are adopted for the SD model. The first scenario that involves estimating stochastic UPD parameters every epoch is called SD1, the second, named SD2, takes the UPD as a time invariant estimated parameter. Figure 3 compares the yaw and pitch angles from the DD and SD1 models. Both the yaw and pitch angles from the DD and SD1 models are nearly identical, which verifies the equivalency of the DD and SD models from the theoretical discussion. Figure 4 shows the results from the DD and SD2 models. The difference in yaw angles between the two models is also very small. The dispersion of the pitch angles from the SD2 model is apparently smaller than that from the DD model, which also supports the conclusions in Section 2.3. The smaller dispersion of the up component of the baseline vector results in a smaller pitch angle dispersion.

The formal uncertainties (FU), standard deviations (Std), and mean values of the three components of the baseline vector (BE, BN, and BU represent the components in the east, north, and up direction, respectively) from the DD and SD models are listed in Table 1. The FUs of BE and BN from all three models are very close. The FUs of BU from the DD and SD1 are about three times as large as that from the SD2 model, which is slightly larger than the theoretical result in Section 2.2, because the theoretical discussion is based on a full upper hemisphere, while the cutoff elevation angle of the real observations is 10°. For the Std value, the differences between BE and BN from the three models are all within 0.04 mm level, whereas, the Std of BU from the first two models are only 1.2 times (about 0.9 mm difference) that of the SD2 model, which is obviously lower than the theoretically predicted ratio value (5.3 times). The most likely cause of this discrepancy is multipath effects, visualized by the low frequency variations in Figure 4b. The validation for the multipath influence will be presented in the next paragraph. For mean values, the differences between the SD2 and the other two models is also larger in the up direction than in the east and north directions, probably also affected by the multipath effect. 

**Figure 5:**
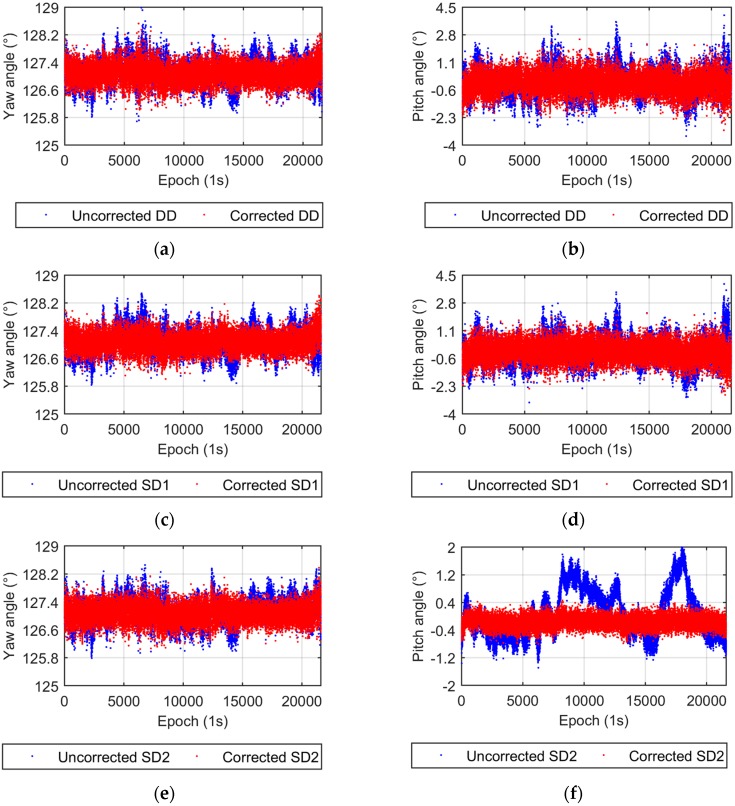
Yaw and pitch angles from the DD and SD models before and after multipath correction. (**a**) Yaw angles from the DD model; (**b**) Pitch angles from the DD model; (**c**) Yaw angles from the SD1 model; (**d**) Pitch angles from the SD1 model; (**e**) Yaw angles from the SD2 model; (**f**) Pitch angles from the SD2 model.

To explain the abnormal large vertical Std value in the SD2 experiment in Table 1 and to get more accurate measurements, the Multipath Hemispherical Map (MHM) approach [28] is applied to mitigate multipath errors. This MHM approach is based on the spatial repeatability of multipath effects under an unchanged environment and is suitable for this static experiment. The attitude solutions from three models before and after the multipath correction are shown in Figure 5. The time series of yaw and pitch angle solutions become flat after multipath correction, which indicates that multipath effects (low frequency part) [29] have been effectively eliminated. 

Since FU relates only to the satellite-antenna geometry and not to the actual errors, multipath correction does not alter the FU values. Thus only Std and mean values are given in Table 2. After multipath correction, the Std values from the DD and SD1 models are about 3.7 times that from the SD2 model, which is much more consistent with the theoretical value (5.3 times). Uncorrected multipath errors (high frequency part) and the limited orbital coverage on the sky might account for the remaining unexplained ratio. Table 2 also indicates that multipath correction not only reduces the solution scatters significantly, in particular for vertical component, but also makes the dispersion ratio between vertical and horizontal solutions closer to the theoretical expectation. Meanwhile the mean values of the baseline solutions from the three models are more consistent after multipath correction. 

### 3.2. Vehicle Test 

**Figure 6:**
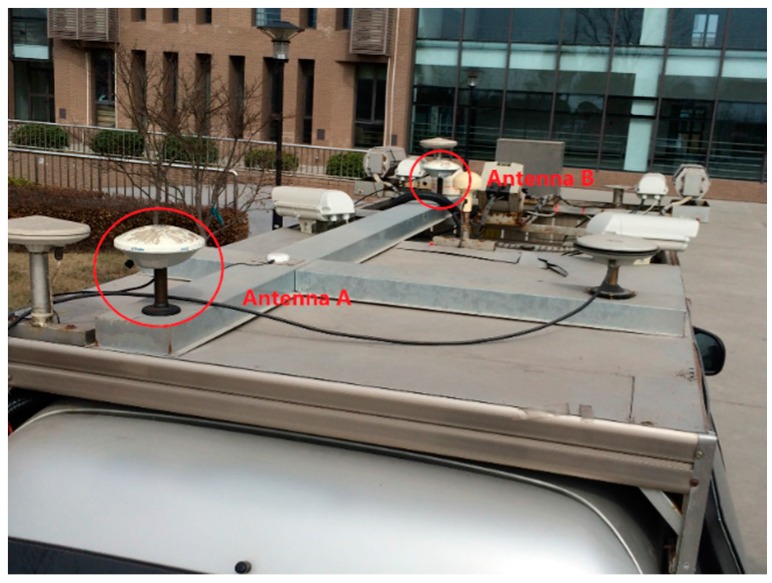
Antenna configurations in vehicle test.

**Figure 7:**
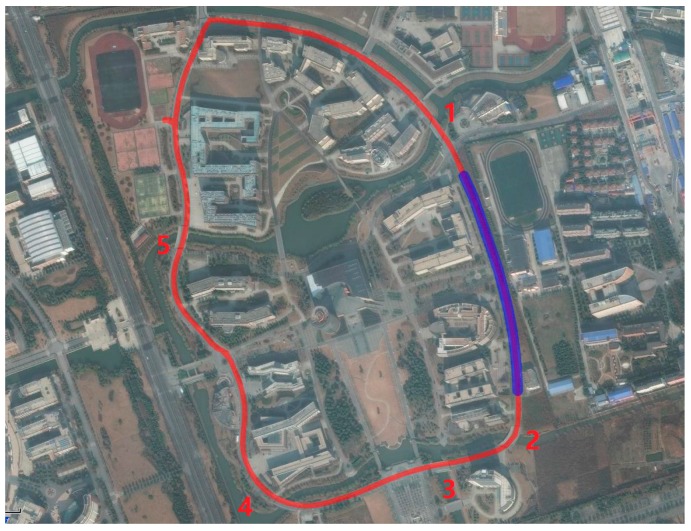
Trajectory of the vehicle. The red line is the whole trajectory, while the blue line represents the road section where the satellite visibility is poor. The five bridges are annotated with numbers.

**Figure 8:**
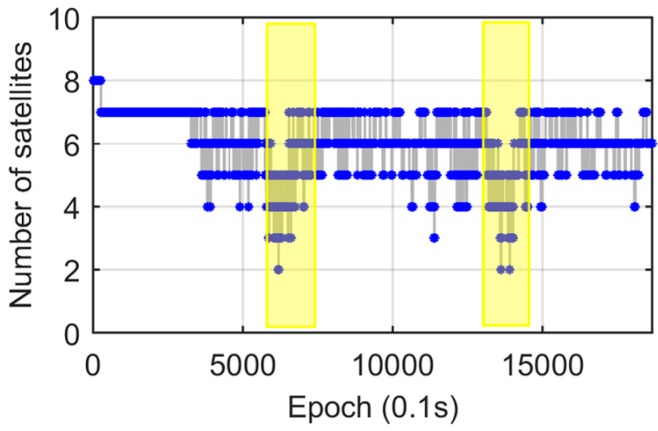
Number of visible satellites with time. Two spans with poor satellite visibility are marked with yellow bars.

A vehicle test was conducted in the campus of East China Normal University on 23 March 2015. Two antennas were mounted on the top of a car (Figure 6) with the baseline parallel to the major symmetric axis of the car. The baseline length was about 2.10 m. The sampling rate was 10 Hz. During the experiment, the vehicle ran along an inner circular route of the campus twice. The trajectory of the vehicle is given in Figure 7, and the five bridges passed by are marked with numbers. The satellite visibility was relatively good (five to seven satellites) most of the time, except for two short spans, when the visible satellite number was reduced to three or less due to surrounding buildings (Figure 8). Statistics indicated that the epochs with more than three satellites accounted for 99% of the total time duration. Note that the DD and SD1 models require a minimum of four satellites to obtain the baseline solutions and the SD2 model requires a minimum of three satellites.

In this test, we compared the three models in kinematic mode from two aspects:

(1) Dispersion of solutions

**Figure 9:**
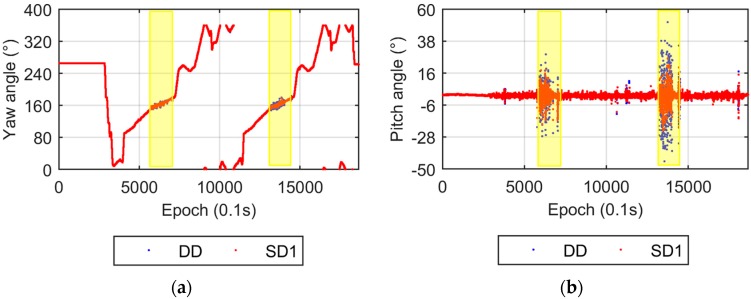
Yaw and pitch angles from the DD and SD1 models. (**a**) Yaw angles; (**b**) Pitch angles. The time periods when the pitch angles with poor accuracy are marked with yellow bars, corresponding to the annotated epochs in Figure 8.

The attitude solutions from the DD and SD1 models are given in Figure 9. They suggest that these two models agree very well in yaw and pitch angle estimation except for the two short spans with poor satellite visibility (highlighted with yellow bars). The two circular laps are embodied by the yaw angle solutions, which coincide with the travel route. 

**Figure 10:**
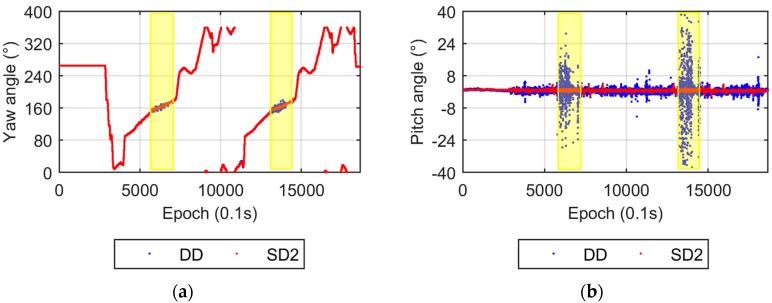
Yaw and pitch angles from the DD and SD2 models. (**a**) Yaw angles; (**b**) Pitch angles. The time periods when the pitch angles with poor accuracy are marked with yellow bars.

**Figure 11:**
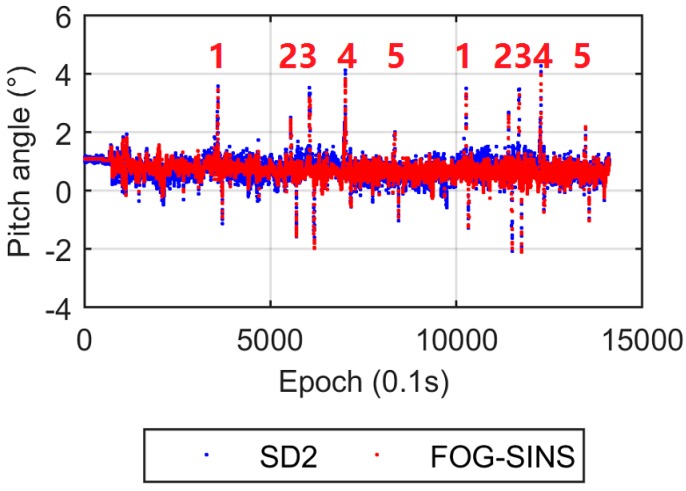
Pitch angles from the SD2 and FOG-SINS in the stage-2 experiment.

The comparisons of attitude results between the DD and SD2 models are shown in Figure 10. The yaw angles from the two models are also in good agreement, while the pitch angles show significant differences. The five bridges can be distinguished easily from the pitch angle solutions of the SD2 model (Figure 10b), while the bridge information appears invisible in the noisy pitch angle series from the DD results. To verify the reliability of the pitch angle solutions from the SD2 model, we performed a supplemental test (called stage-2 test). In the stage-2 test a strapdown inertial navigation system-based fiber optic gyroscope (FOG-SINS), a KY-INS300 from BDStar navigation Company was introduced to provide the accurate attitude solutions. Its sampling rate was 100 Hz. The same car with the same two mounted antennas made two laps along the same campus route. Comparison between the pitch angles from the SD2 and FOG-SINS solutions in stage-2 is given in Figure 11. These two results are in good agreement and the five bridges can be identified from both results clearly.

**Figure 12:**
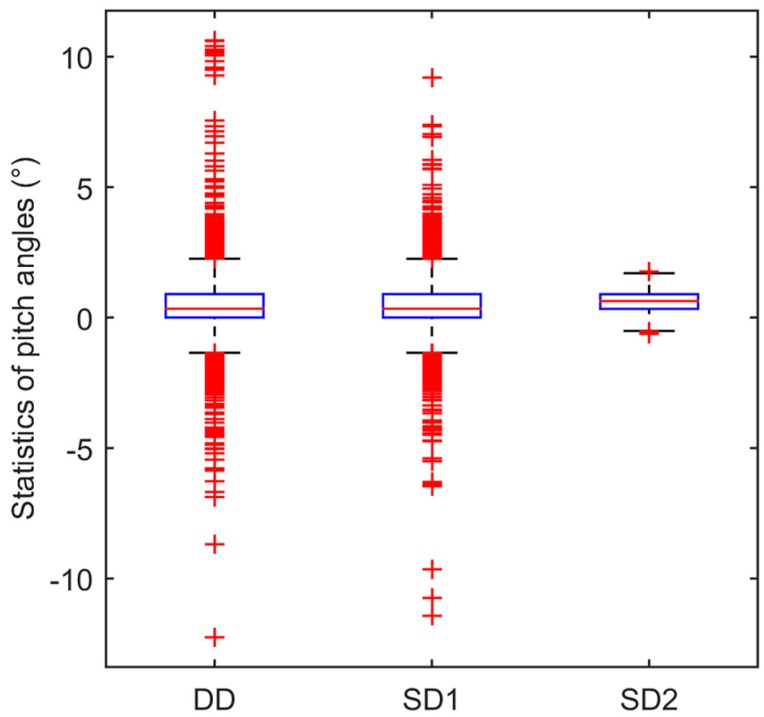
Boxplot of pitch angles from the three models.

To assess the dispersion level of the pitch angle solutions quantitatively, boxplots [30] of the pitch angle estimates from the three models are given in Figure 12. We exclude the epochs when the vehicle went across the bridge and encountered poor satellite visibility (annotated by yellow bars in the above figures), so that at the remaining epochs the route can be considered as flat and the pitch angle should be a constant and close to zero. In Figure 12 the red line inside the blue box represents the median value (percentile 50th), the upper and lower boundaries of the blue box represent the upper quartile (Q1: percentile 25th) and lower quartile (Q3: percentile 75th) respectively, the upper and lower whiskers represent the threshold values which distance to the upper and lower quartile is 1.5 × (Q3 − Q1), the solutions outside the whisker are considers as outliers. The results from the DD and SD1 are very close. The differences between median, Q1, Q3, upper whisker, and lower whisker from the DD and SD1 are all no more than 0.05°. It suggested that these two model are also equivalent in kinematic mode. In addition, compared with the above two models, the SD2 model is more sensitive and robust. We used the range between upper and lower whiskers to access sensitivity, the value from the DD and SD1 models is about 3.7° while that from the SD2 model is 2.2°, which tells why it’s easy to detect the period when the vehicle went across the bridge with the SD2 model. Maximum and minimum values are used to measure the robustness. The maximum and minimum pitch angle values from the SD2 are 1.8° and −0.6°, while those from the DD and SD1 models are about 10° and −11°.

(2) Sensitivity to the satellite availability

**Figure 13:**
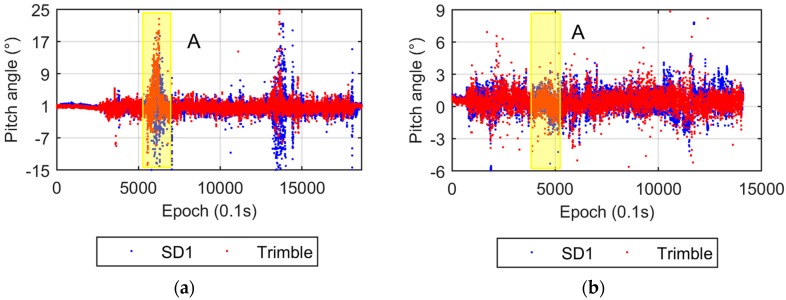
Pitch angles from the SD1 model and Trimble. (**a**) Original; (**b**) Stage-2. The first abnormal time period is labeled with “A”.

In Figure 9, there are two time periods when both yaw and pitch angle solutions from the DD and SD1 models suffer poor accuracy (highlighted by yellow bars). During these two periods the vehicle passed the same road section where both sides were surrounded by buildings (blue line in Figure 7) and the satellite visibility was poor (Figure 8). To verify this results, we also provide the real-time pitch angle outputs from the Trimble BD982 GNSS receiver in Figure 13a. It shows a similar pattern as our SD1 results. Conversely, the results from the SD2 model are very stable all the time (Figure 10).

**Figure 14:**
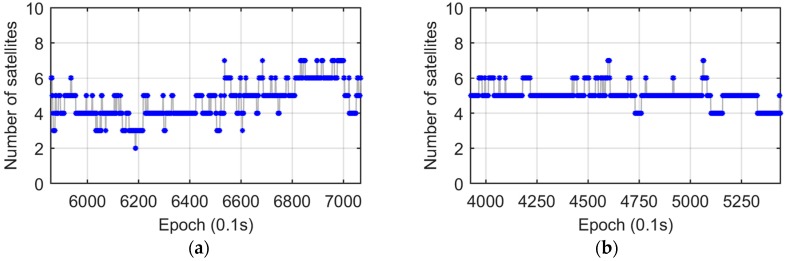
SD observables during period A. (**a**) Original; (**b**) Stage-2.

The results of the stage-2 experiment show that no obvious outliers from both the SD1 and Trimble solutions (Figure 13b) in the abnormal period A (highlighted by a yellow bar and labeled with “A” in Figure 13), which is contrary to the original results (Figure 13a). Note that in the stage-2 test the car drove faster than in the original test, so that its time span was slight shorter. To reveal the reason, a comparison between observations is performed. Figure 14 shows the SD observables from both stages during time period A. Obviously, stage-2 (Figure 14b) has a larger number of satellites than stage-1 (Figure 14a). It indicates that the DD and SD1 models are more sensitive to the satellite visibility, and SD2 model is superior in AD under sheltered environments, such as urban canyons.

## 4. Conclusions

The emerging multi-antenna synchronized GNSS receiver uses a common clock for the multiple antennas. Its SD observables are able to eliminate both satellite and receiver clock errors simultaneously. Such an advantage has encouraged scientists to reconsider the equivalency problem between the SD and DD schemes and to explore the possibility of further improving the accuracy of AD using this type of receiver [20,21]. 

Theoretical discussions and real static and kinematic experiments demonstrate that if estimating clock parameters using the SD observables (non-common clock scheme), the SD and DD models are equivalent for both formal uncertainties and dispersions of baseline solutions even using this type of receiver. If only estimating UPD parameters using the SD observables (common clock scheme), however, the two scenarios shows different results. If we estimate stochastic UPD parameters every epoch, this SD model (called SD1) is also equivalent to the DD model, whereas if we estimate only one UPD parameter for all epochs or take it as a known constant, this SD model (called SD2) and the DD model are no longer equivalent. Further investigations indicate that the formal uncertainties and dispersions of the horizontal baseline components of the SD2 model are only slightly smaller than those of the DD model. For the vertical component of baseline solutions, however, the formal uncertainties of the SD2 model are two times smaller than those of the DD model, and the solution dispersions of the SD2 model are even more than twice smaller than those of the DD model, so the yaw angle solutions from the SD2 and DD models are basically the same, but the accuracy and quality of the pitch angle solutions from the SD2 model are significantly improved compared to that from the DD model. Furthermore, the SD2 model requires a minimum of three satellites to obtain the baseline solutions, while the DD model requires a minimum of four satellites. Such a difference makes the SD2 model much more attractive for AD in environments with poor satellite visibility.

Our kinematic experiments showed that with the SD2 model the GNSS short baseline (2 m) technique is able to catch even tiny events of 1.5°–3° pitch angle variations, while the pitch angle solutions from the DD model seems too noisy to reach this accuracy level. Such progress certainly extends the horizon of GNSS applications, for example for unmanned vehicles and aircraft. To take full advantage of the multi-antenna synchronized GNSS receiver, the SD2 model must be adopted. The SD2 model also imposes new challenges for GNSS data analysis. For the SD2 model the ambiguities are no longer integers because the ambiguity parameters and UPD parameter are highly correlated. There are many proposed approaches to resolve the integer SD ambiguities [23,24,31], so we will not go into details in this paper.

## Figures and Tables

**Table 1:** FU, Std, and mean values of each component of the baseline vector from DD, SD1, and SD2 models (baseline length = 398 mm).

	FU			Std			Mean		
	DD	SD1	SD2	DD	SD1	SD2	DD	SD1	SD2
BE (mm)	2.279	2.279	2.206	2.350	2.350	2.315	316.832	316.832	316.823
BN (mm)	2.642	2.642	2.506	2.404	2.404	2.423	−239.761	−239.761	−239.826
BU (mm)	5.742	5.737	1.882	5.639	5.635	4.725	−0.730	−0.729	0.556

**Table 2:** Std and mean values of each component of the baseline vector from the DD and SD models after multipath correction (baseline length = 398 mm).

	Std			Mean		
	DD	SD1	SD2	DD	SD1	SD2
BE (mm)	1.696	1.696	1.672	317.176	317.176	317.188
BN (mm)	1.916	1.916	1.841	−239.648	−239.648	−239.612
BU (mm)	4.145	4.140	1.109	−1.236	−1.235	−1.122
